# In Vitro Modulatory Effect of Stevioside, as a Partial Sugar Replacer in Sweeteners, on Human Child Microbiota

**DOI:** 10.3390/microorganisms9030590

**Published:** 2021-03-13

**Authors:** Florentina Gatea, Ionela Sârbu, Emanuel Vamanu

**Affiliations:** 1 Centre of Bioanalysis, National Institute for Biological Sciences, 296 Spl. Independentei, 060031 Bucharest, Romania; florentina.gatea@incdsb.ro; 2 Department of Genetics, University of Bucharest, 36-46 Bd. M. Kogalniceanu, 5th District, 050107 Bucharest, Romania; ionela24avram@yahoo.com; 3 Faculty of Biotechnology, University of Agronomic Science and Veterinary Medicine, 59 Marasti blvd, 1 District, 011464 Bucharest, Romania

**Keywords:** pattern, butyric acid, DPPH, diabetes, *Lactobacillus*

## Abstract

The effect of stevioside on human health is still insufficiently highlighted by recent research. The total or partial replacement of sugar with sweeteners influences the general state of health, especially the human microbiota’s response as a determining factor in the onset of type 2 diabetes. The present study aimed to present the long-term (one-year) in vitro effect that regular stevioside consumption had on children’s pattern microbiota. A metabolomic response was established by determining the synthesis of organic acids and a correlation with antioxidant status. An increase in the number of bacterial strains and the variation of amount of butyrate and propionate to the detriment of lactic acid was observed. The effect was evidenced by the progressive pH increasing, the reduction of acetic acid, and the proliferation of *Escherichia coli* strains during the simulations. Synthesis of the main short-chain fatty acids (SCFAs) was interpreted as a response (adaptation) of the microbiota to the stevioside, without a corresponding increase in antioxidant status. This study demonstrated the modulatory role of stevioside on the human microbiota and on the fermentation processes that determine the essential SCFA synthesis in maintaining homeostasis. The protection of the microbiota against oxidative stress was also an essential aspect of reducing microbial diversity.

## 1. Introduction

A microbial community’s reaction to the administration of a product is one of the most dynamic chapters of the human microbiota study [1]. This ecosystem’s plasticity is one of the critical factors that ensure the role of buffers in the action of various exogenous factors [2]. The incidence of degenerative pathologies was explained, in recent studies, by changing the microbial pattern and increasing oxidative stress. Dysfunctions are favored byproducts promoted in modern man’s diet and consumed regularly and are related to an emotional state. The gut–brain axis plays an essential role in supporting and controlling physiological functions [3].

A category of chemical compounds present in the diet of healthy people but especially in that of people with various metabolic disorders (e.g., obesity, diabetes, cardiovascular diseases) is that of sweeteners. Although their use as sugar substitutes is a common practice justified by attempts to reduce caloric intake, many of their effects on health remain little known due to the lack of long-term studies on human volunteers, frequent consumers of these compounds. Due to a variety of factors that may influence this type of research (e.g., type of diet, physical activity, health, drug treatment, microbiome health, co-use of several types of sweeteners), it is difficult to assess the true impact of each sweetener on health. Competent authorities approve the use of these sweeteners, but despite this, controversies regarding their safety and health benefits exist [4]. In this context, the European Food Safety Authority decided to re-evaluate sweeteners as food additives, developing protocols on hazard identification and the characterization of sweeteners.

In recent years, a new trend, that of using natural food, has influenced diet choices. Natural sweeteners such as sugars (fructose, trehalose, and tagatose) sugar alcohols (maltitol, mannitol, sorbitol, xylitol, erythritol, isomalt, and lactitol), proteins (thaumatin, brazzein, mabinlin, monelin), and terpenoid glycosides (stevioside, rebaudioside A, glycyrrhizin, or glycyrrhizic acid) are increasingly being used [5].

These natural sweeteners, stevioside, have enjoyed special attention, justified both from the economic point of view and the potential health beneficial effect. There are numerous studies on the beneficial effect of stevioside. These report the antioxidant, antimicrobial, and anti-inflammatory potential of stevia, as well as the antihyperglycemic and hypolipidemic effects of stevioside and its anticancer effect, reduction in systolic blood pressure or improved lipid profile, glycemic control, or possible positive physiological effects on the liver [4,5,6,7,8,9,10,11]. However, it can be seen that a common feature of these studies is that they are performed on people who already have various health problems and are primarily based on the study of the effect of stevioside on physiological parameters [12]. In comparison, fewer data refer to the influence of long-term stevioside use on healthy people and the large intestine microbiota [13].

Current research aims to assess the effects of increased sugar consumption in products considered to be dietary [14,15]. Thus, some data indicate adverse effects, such as loss of intestinal motility, stimulation of inflammatory processes, or cancer risk [5]. Particular attention was paid to non-nutritive sweeteners’ role in the long-term change in the human microbiota pattern (dysbiosis). This aspect results from the insufficient knowledge of the metabolic syndrome mechanisms that lead to the installation of degenerative pathologies: hypertension, obesity, diabetes, or proinflammatory processes—cytokine synthesis [16,17]. These can cause irreversible changes in physiological functions, leading to neurodegeneration.

Understanding the processes related to microbiota and stevioside interaction is topical research due to the widespread consumption of sweeteners [17]. These compounds considered toxicologically safe have not an impact study for long-term use [18]. The simulators can explain the effect of compounds reaching the colon and how the microbiota structure reacts microbiologically and metabolomically in vitro. There can be tested the impact under different conditions, variations of the administered doses, bioavailability, or other physiological parameters that will provide an image of the effect in vivo, without involving studies on human subjects [19]. The use of low-capacity laboratory simulators is a current trend due to the flexibility and ability to track the evolution of many bacterial compounds and strains in real-time. Other advantages that may result from such a study are the possibility to perform the same simulation in parallel (reproducibility) and to track the bioavailability of some target compounds (e.g., stevioside) [20].

The information provided by these in vitro experiments was limited by the lack of interactions with the epithelial barrier, which, in addition to secreting important immune mediators and delivering bacterial antigens, played an essential role in the gut microbiota–host symbiotic relationship [21]. In this context, it was also difficult to assess the influence of important metabolites (produced in vivo) such as indoles (which limited the decline of the endothelial barrier due to age and acted in the situation of inflammatory responses to acute stressors) or secondary bile acids (which could regulate the composition of the microbiota) [22,23,24,25,26,27,28]. Other important metabolites frequently associated with the anti- and proinflammatory response or dysbiosis were A-lipopolysaccharide (A-LPS, produced by *Bacteroidetes*) and P-lipopolysaccharide (P-LPS, known as endotoxin and produced by Gram-negative bacteria) [29,30,31].

The purpose of this study was to test the effect that regular consumption of stevioside has in vitro, on the pattern of children’s microbiota over a year. The microbiota analysis was performed by qPCR, and the samples were analyzed monthly, except for the first month of consumption when a weekly analysis was performed. The synthesized acid pattern was also determined (metabolomic study), especially SCFAs, and the evolution of pH values. The data were correlated with the antioxidant status analysis in response to regular stevioside consumption.

## 2. Materials and Methods

### 2.1. In Vitro Fermentation Model

#### 2.1.1. Fecal Samples

The microbiota from healthy children (minimum three samples from three different persons) was reconstituted from the feces, according to the ethical guidelines of UASVM Bucharest (ColHumB Registration number: 1418/23 November 2017; http://gissystems.ro/colhumb/ (accessed on 1 January 2020). Individual samples were obtained from persons who had not received treatment with antibiotics or any other interfering drugs over the past six months, as these agents could alter the microbiome fingerprint. All the samples were collected in 10% glycerol and stored at −75 °C until use. The inoculum was realized by using peptone water over a 7-day stabilization period until its use for the in vitro simulations (fresh microbiota pattern) [32].

#### 2.1.2. Simulation Conditions

All in vitro tests were performed in a modified version of the GIS1 simulator. The simulations were performed via Phase 2 transit simulation through the colon in the unicompartmental system (http://gissystems.ro/gis-technology/ (accessed on 1 January 2020). The modified version consists of the introduction in the simulation bottle (Duran laboratory bottle, 1 L capacity) 4 mg/kg body weight stevioside [33], consumption two days (commercial form), dissolved in NaCl 0.9%. Thus, after two days, the old bag was removed, and a new one was inserted in sterile conditions. The samples were obtained each week in the first month and one time per month every 30 days. The samples were centrifuged in sterile conditions, and the supernatant was kept at −15 °C for metabolomic analysis. The sediment was mixed with glycerol 10% and maintained at the same temperature for qPCR analysis [34]. The number of replicates (in vitro simulations) done was three.

### 2.2. Determination of Microbiota Pattern

One milliliter of each sample was used for DNA extraction by applying the PureLink Microbiome DNA Purification Kit (Invitrogen, Waltham MA, USA). The DNA concentration and purity were determined by reading the absorbance of NanoDrop 8000 spectrophotometers (Thermo Fisher Scientific, Waltham MA, USA). The primers coverages were analyzed in the Arb-SILVA database (https://www.arb-silva.de/ (accessed on 10 January 2020). An amount of 5 ng of DNA was introduced in each reaction. The other PCR amplification conditions, including primer details, were presented in a previous study [32].

### 2.3. Determination of Metabolomic Response

Separation and quantitative determination of short-chain fatty acids (SCFAs) from samples representing microbial cultures were performed using a previously developed and validated method [35]. Before analysis, samples were centrifuged, filtered on 0.2 µm membranes (Millipore, Bedford, MA, USA), and degassed.

### 2.4. Determination of Antioxidant Response

*In vitro* antioxidant response to stevioside consumption was evaluated by three methods: the total antioxidant activity [36], antiradical activity by DPPH scavenging activity [37], and inhibition of lipid peroxidation by using the egg-yolk homogenates as substrate [38].

### 2.5. Statistical Analysis

All the parameters investigated were evaluated in triplicate, and the results were expressed as the mean, standard deviation (SD) values of three observations. The mean and SD values were calculated using the IBM SPSS Statistics 23 software package (IBM Corporation, Armonk, NY, USA). The significance level for the calculations was *p* ≤ 0.05. The differences were analyzed by ANOVA, followed by a Tukey post hoc analysis. The experimental data analysis and correlation were done with the IBM SPSS Statistics software package (IBM Corporation, Armonk, NY, USA) [32]. Principal component analysis (PCA) was performed using GraphPad Prism 9.0.2. (San Diego, CA, USA).

## 3. Results

The in vitro simulation determined the effect of regular stevioside consumption on children’s microbiota. The microbiota pattern was assessed by qPCR. Modulation of the metabolomics pattern was determined by evaluating the amount of SCFAs and lactate over one year. These data were also correlated with the antioxidant status in response to this sweetener’s continued and constant consumption.

### 3.1. The Response of the Microbiota Pattern after Stevia In Vitro Consumption

**Figure 1:**
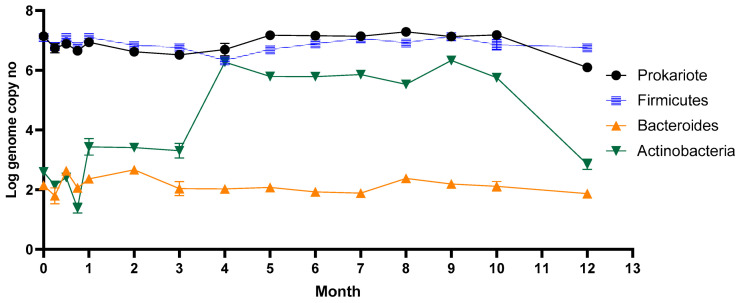

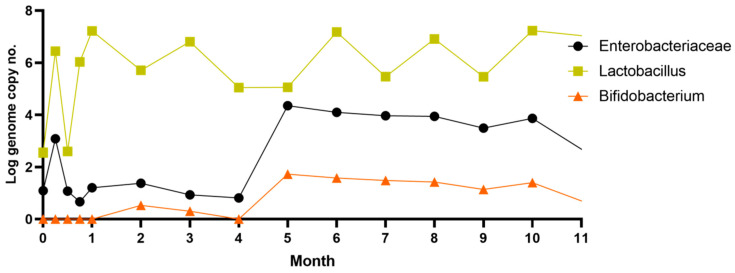
Long-term stevia effect on the main groups of bacteria after in vitro long-term stevioside consumption.

The microbial dynamics following stevioside consumption during the year are represented in Figure 1. The gut microbiota is a complex environment dominated by bacterial strains. Each individual can host up to 300 different species in their gut. *Firmicutes*, which was the dominant phyla from the human gut, was maintained in a high number, around 10^7^ genome copy no./mL, during long-term stevia administration. *Actinobacteria* instead, after three weeks, started to grow considerably, reaching a maximum of 106 cells/mL (*p* ≤ 0.05; r = 0.6099) within 16 weeks and remained constant for half a year. The number of *Actinobacteria* decreased after ten months, reaching the initial number of cells. Stevia consumption did not affect the *Bacteroides* group, which remained constant during the entire year.

The number of lactobacilli varied from one week to another, reaching a maximum of 107 copies no./mL after only one month. Throughout the period, the number of lactobacilli remained relatively high despite the variation. Our results are similar to data previously reported by a study on rebaudioside A’s influence on rats’ gut microbiota after 28 days of oral administration [39]. This study reveals that low doses of rebaudioside A cause slight changes in lactobacilli diversity, while high doses cause a significant increase in those species. The number of *Enterobacteriaceae* and *Bifidobacteria* increased significantly after four months (*p* ≤ 0.05; r = 0.5649), a week later after *Actinobacteria*.

Given that *Bifidobacterium* was part of the *Actinobacteria* phylum, its consecutive growth can be explained. In addition to bifidobacteria, this phylum includes other members, such as *Corynebacterium*, *Actinomyces*, *Rothia*, and *Propionibacterium*, known to colonize the intestinal tract of healthy people. Thus, the consumption of stevia caused the growth of other members from the *Actinobacteria* phylum.

### 3.2. The Response of the Metabolomic Pattern after Stevia In Vitro Consumption

**Figure 2:**
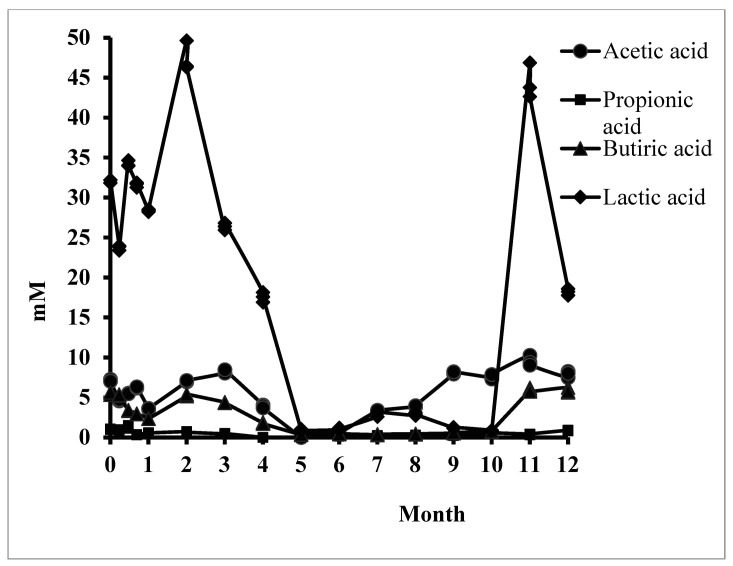
The amount of acetic, propionic, butyric, and lactic acids after in vitro long-term stevioside consumption.

Figure 2 shows the evolution of the three key SCFAs and lactic acid as the main organic acid resulting from the in vitro simulation. The average molar ratio (3:1:1) between SCFAs [40] was severely disrupted following regular stevioside consumption. The significant decrease in the synthesis of these acids that were identified in the middle of the consumption period could be recovered for acetic and butyric acid for up to one year. The result was correlated with a change in the fermentative behavior of the microbiota. The metabolic rate caused a decrease in lactic acid and propionic acid synthesis.

Butyric acid, the most important SCFA, was synthesized continuously, but a significant decrease was recorded from Month 5 to Month 10. The microbiota regained its synthetic capacity after 11 months of in vitro simulation (528.28 ± 16.33 µg/mL) compared to the control sample. This result was relevant to the colon’s physiological function, but the dysbiotic microbial pattern induced a report of SCFAs, which explains the long-term implications for human health.

**Figure 3:**
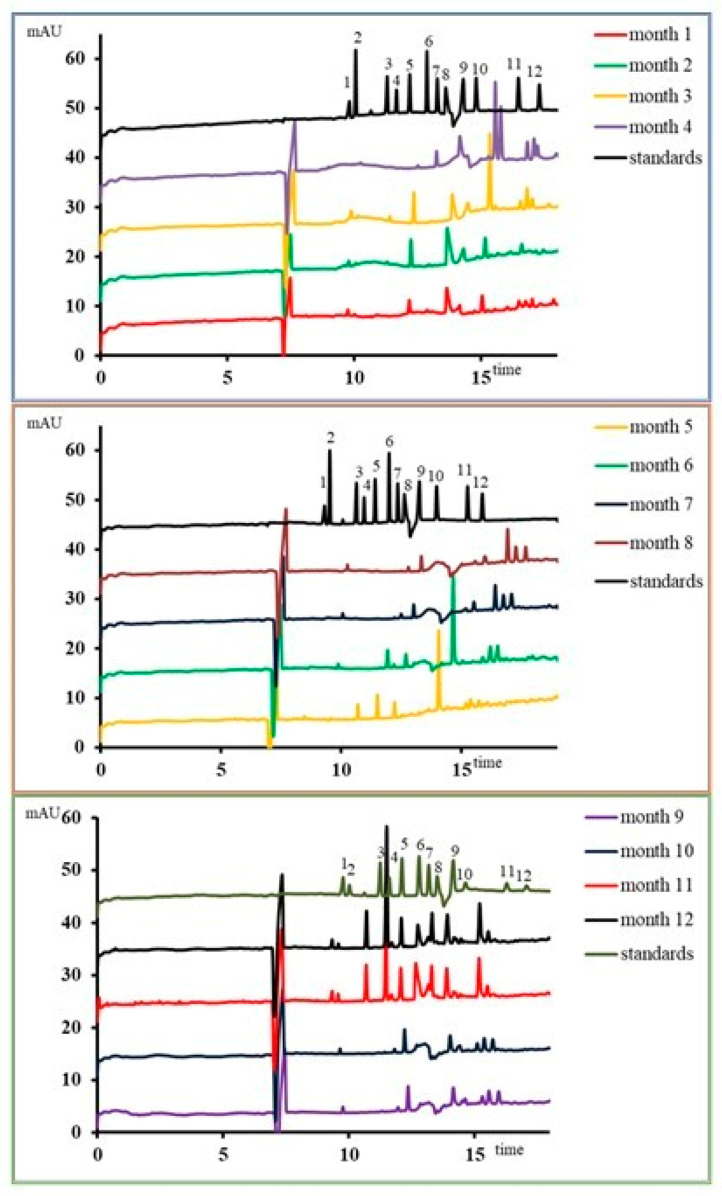
The amount of acetic, propionic, butyric, and lactic acids after in vitro long-term stevioside consumption. Standards: 1—formic acid; 2—oxalic acid; 3—succinic acid; 4—malic acid; 5— tartaric acid; 6—acetic acid; 7—propanoic acid; 8— butyric acid; 9—lactic acid; 10—benzoic acid; 11— phenyllactic acid; 12—OH phenillactic acid.

The other organic acids (Figure 3) showed the same dysbiotic pattern, which was influenced by the microbiota’s metabolic function. By the middle of the consumption period, a unitary pattern was determined compared to the control. After 4–6 months of consumption, some acids (malic acid) were no longer intermittently synthesized. During the same period, an abnormal increase of benzoic acid was recorded (27.70 ± 0.16 µg/mL, *p* ≤ 0.001). Stevioside also induced general increases in hydroxyphenyllactic acid compared to the control sample. Overall, stevioside reduced the metabolic processes of organic acid synthesis and disrupted the initial equilibrium state.

### 3.3. Antioxidant Status after Stevia In Vitro Consumption

**Figure 4:**
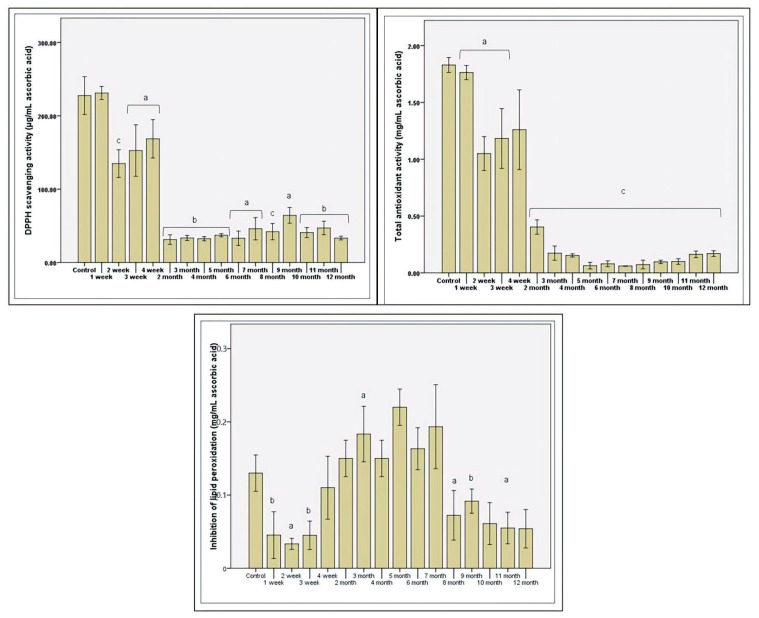
The antioxidant potential after in vitro long-term stevioside consumption. Different letters represent. significant statistical differences (control vs. samples; *p* ≤ 0.05), n = 3; fresh microbiota was used as control.

Antioxidant status was assessed in vitro by three methods by the microbiota’s response to various factors that support oxidative stress. In the first month, the antiradical response maintenance was determined and decreased after the second month (*p* ≤ 0.01; Figure 4). The functional plasticity and gradual consumption of stevioside induced a slight increase in the antioxidant response toward the end of the first month of administration (Figure 4). Protection against free radicals was minimal in the middle of the study, which was in contrast to the increase in protection against lipid peroxidation. The maximum protection at the lipid layer was calculated in the fifth month (over 0.2 mg/mL equivalent ascorbic acid). After this interval, the trend was downward, in line with antiradical protection. The total antioxidant status was low, with the increase in the period of consumption of stevioside (Figure 4).

The gradual loss of total antioxidant protection resulted from the disrupted fermentative activity with the regular consumption of stevioside. Thus, decreased production was correlated with decreased total antioxidant status, demonstrated in previous studies [41]. The accumulation of toxic molecules due to the dysbiotic status determined a decreasing antioxidant reaction characteristic of the presence of nitrite. Microbial paternal cells’ response has increased protection against lipid peroxidation due to the decreased microbial diversity and decreased metabolic activity of favorable strains, such as those of the genus *Lactobacillus*.

## 4. Discussion

Eating habits and the increased consumption of sweets have led to the development of new theories related to nutrition and the marketing of products that reduce food excesses’ negative impact. There was a need to identify natural sources of nontoxic sweeteners. Stevia is considered safe, being extracted from *S. rebaudiana*, but the long-term impact is still insufficiently understood.

The effects of stevioside-based product consumption on the microbiota can only be determined from applied studies. Daily analysis of *Lactobacillus* strains resulted in a decrease in their number in the first week of use (Supplementary Figure S1). These data were also supported by an increase in the pH value, maintained after the first month of simulation. If the values varied a lot in the first week due to the microbiota pattern changes, in the second week, the values started to balance (*p* ≤ 0.01; Supplementary Figure S2). An adjustment of the fermentative behavior determined results, while other carbon sources were still in the simulated environment (e.g., glucose). The modulation of the fermentative behavior was also confirmed by reducing lactic acid synthesis (Figure 3), which was also supported by the gradual increase of pH after the first month. These data support previous studies that have shown that changing the pH value in the colon influences *Akkermansia muciniphila*, which has the effect of lowering glucose tolerance [42], one of the causes of type 2 diabetes [43].

The results obtained reveal that the *Bacteroides* group responsible for the hydrolysis of stevioside and rebaudioside A to steviol [44] did not show significant numerical variations during the simulation. The accumulation of steviol in the system (which normally was absorbed from the intestine and is eliminated by biliary excretion in vivo) may be a possible explanation for slight variations in the number of cells recorded for other/some types of bacteria. This hypothesis was supported by new research demonstrating the inhibitory effect of a stevia herbal supplement on bacterial communication regulating microbial behavior [45].

The data obtained demonstrated the modulating role of stevioside in the bacterial metabolism of children’s microbiota. Like polyphenols [46], stevioside stimulated specific bacterial groups’ proliferation, altering the metabolomic signature. No antimicrobial action was observed, but only a reduction in the number of microorganisms. Modulation at the metabolomic level was characterized by the presence of large amounts of specific organic acids. The gradual decrease of propionate caused the most critical change in the metabolomic pattern. This influenced the metabolomic balance, leading to physiological dysfunctions most often associated with type 2 diabetes [47].

The metabolomic signature was an indicator of microbiome balance disturbance [40]. These data confirmed the role of *Actinobacteria* in developing obesity by suppressing propionic acid synthesis (Figure 2). As part of the critical SCFA for human wellbeing, propionic acid is also a biomarker in the early onset of obesity, an important issue for young people. The decrease in the number of bifidobacteria is an indicator of the onset of diabetes, an aspect demonstrated in previous studies [48]. In this study, the intestinal microbiota and the metabolomic signature showed a unitary system, which established regular stevioside consumption’s modulatory function.

**Figure 5:**
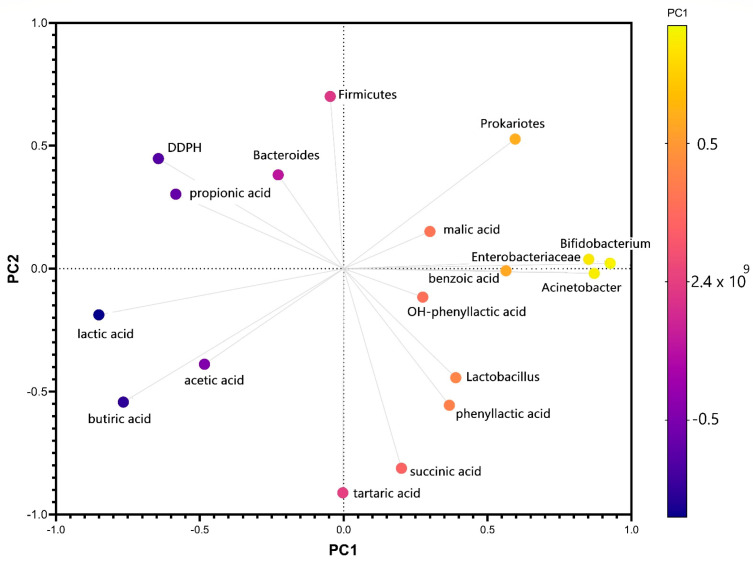
Biplot for the first two principal components of PCA obtained using the gut microbiota, metabolic activity, and antiradical potential variation after in vitro long-term stevioside consumption.

The first principal component has large positive associations with *Actinobacteria*, *Bifidobacteria*, *Enterobacteriaceae*, and benzoic acid, while the second component has large negative associations with butyric, tartaric, and succinic acids production. The variation in the number of *Bacteroides* strains was correlated after PCA analysis with the value of antiradical potential (Figure 5) and with the amount of propionic acid, which confirmed the data of a previous study that demonstrated the relationship between diabetes and the pattern of favorable strains in the microbiota [49]. The same analysis showed a negative correlation between propionic acid and *Actinomycetes*. It has also been demonstrated in vitro that, although there are differences from one interval to another, the effect of stevioside consumption results from interaction with the tested microbiota. The microbiota’s plasticity was one of the main endogenous factors that reacted to long-term exposure to stevioside. The metabolomic signature determined in vitro was negative because it cannot fully correlate the bacterial metabolism with the cometabolic pathway mentioned in vivo studies [50]. The PCA score plots obtained using HPLC and RT-PCR data are displayed in Supplementary Figure S3, using the first two principal components, which covered 36.51% and 17.92% of the variance (Supplementary Figure S4).

A recent study on six-week-old Wistar rats fed a control or high-fat diet (HFD) by administering sweeteners in water, a recent study on six-week-old Wistar rats fed a control high-fat diet (HFD). It was observed that the gut microbiota was differentially modified by the fat content in the diet and by the type of sweetener [51]. The beta diversity analysis showed that 18.42% of the microbiota variation was due to the type of sweetener. The ratio of *Bacteroidetes*/*Firmicutes* to the steviol glycoside group was 0.3–0.55, indicating a significant alteration in the gut microbiota. The most affected genera were *Lactoccoccus*, *Mucispirillum*, and *Bifidobacterium* in the groups fed noncaloric sweeteners. The presence of fats in the food led to an increase in the genus *Akkermansia* for all sweeteners. They also increased the abundance of *Desulfovibrio*, *Enterococcus*, and *Butyricimonas* genera. At the species level, the abundance of *Faecalibacterium prausnitzii* increased for steviol glycoside administration in the presence or not of HFD. The metagenomic analysis showed that the groups fed with steviol glycosides + HFD had the lowest number of genes involved in LPS synthesis. In contrast, steviol glycosides appear to activate proinflammatory signaling pathways by increasing the expression of TLR4, TNFα, and NF-κB, suggesting a microbial inflammation. Another aspect worth mentioning is that SV and sucralose were the significant producers of acetate, which was associated with the development of hepatic steatosis.

Increasing the number of *Bifidobacteria* was a key element in the synthesis of acetic and butyric acid. The increase in *Enterobacteriaceae* was supported by the increase in pH and the gradual reduction of the molar ratio of the three SCFAs.

The total loss of the three SCFA amounts after the fourth and fifth months of stevioside consumption resulted in a decrease in the total antioxidant potential. A positive aspect was preserving the synthesis of butyric acid, which supported the intestinal epithelium’s integrity, an element correlated with initial resistance to lipid peroxidation (Figure 4), which promoted the dysbiotic state. This aspect could also be explained by the loss of certain genera species in the in vitro study, being one of the study’s negative points compared to in vivo tests, which use animal models. In vivo, SCFAs (which provide a significant percentage of daily caloric energy) are transported through the membrane and metabolized in the colonocytes or other body cells [52]. The gradual elimination from the system could generate new metabolic responses of the microbiota.

The participation of SCFAs in two major signaling pathways involved in metabolic inflammation, glucose, and lipid metabolism is well known [53]. Numerous studies have shown correlations between SCFAs, BMI, body fat distribution, and fat to lean mass ratio in children [54]. While acetate is a precursor in lipogenesis, propionate inhibits the use of acetate in cholesterol synthesis [55,56]; therefore, the concentrations of both propionic and acetic acid are near related to the processes that contribute to the reduction of body fat. Constant administration of stevioside in the system over a year has led to a drastic decrease in the amount of propionic acid in parallel with an increased acetic acid concentration. In vivo, these results may lead to an increase in lipogenesis and/or cholesterol synthesis.

Insulin resistance, as well as cardiovascular diseases, is frequently associated with metabolic inflammation. Studies have shown that metabolic inflammation can be diminished by bioactive compounds present in the diet through the inhibitory effect that they have on TNF-α, the inhibitor of kB kinase involved in activating NF-κB, the central mediator of immune response [57,58,59,60]. The same effect is exhibited by SCFAs that reduce NF-kB activity to their efficiency: butyrate, acetate, and propionate. The low concentrations of SCFAs recorded in the experimental system during stevioside administration can be considered a possible inflammatory factor, but it is not easy to assess whether their biosynthesis would be the same when other biologically active compounds were present in the medium. Therefore, new experimental data are needed to clarify these mechanisms and their effect on the health of both the colon microbiota and the whole body.

The increase in the concentration of phenolic acids such as benzoic acid, hydroxyphenyllactic acid, and phenyllactic acid can be correlated with the system’s antioxidant status, respectively, with the activity of bacterial strains such as *Bifidobacteria* and *Lactobacilli*. While benzoic acid has a prooxidant effect, hydroxyphenyllactic acid and phenyllactic acid play an important role in lowering free radical concentration at the cellular level by acting as natural antioxidants [61]. These phenylcarboxylic acids appear to have bioregulatory effects both in the microbiota and whole organisms [62,63]. Although elevated levels of phenolic acids such as benzoic acid, hydroxyphenyllactic acid, and phenyl lactic acid could not be directly correlated with bacterial strains of the microbiota in the experimental system, they may be indicators for future in vivo studies on the effect of stevioside on the microbiota. These claims are supported by research linking the microbiota and phenyl carboxylic acid levels with hepatic steatosis and fibrosis [64], severity and mortality in critically ill patients [62], and sepsis [65].

Among the metabolites determined in our experiments is succinic acid, the concentration of which can be analyzed in terms of its synthesis by certain bacteria (*Bacteroidetes*, *Actinobacteria*, and the Negativicutes class of *Firmicutes*, etc.) but also its consumption in the system (*Bacteroidaceae, Acidaminococcaceae, Veillonellaceae, Ruminococcaceae*) [66]. The concentration of succinic acid in the system had extreme variations starting from relatively constant concentrations during the first month of the study, decreasing until the total absence, and then reaching high concentrations toward the end of the study period. No positive correlation has been established between the level of succinic acid concentration and the dynamics of developing a particular bacterial strain. However, the role of succinic acid in the system cannot be minimized when we consider the use of stevioside in diabetes or obesity. A diet enriched with succinate-synthesizing bacteria (*Prevotellacopri*) in mice has led to improved glucose metabolism [67].

## 5. Conclusions

The constant and long-term administration of stevioside has had a modulating role on the microbiota and the metabolomic fingerprint. Stevioside has shown that it cannot be used exclusively as a sweetener because it has induced structural and functional changes in the microbiota, decreasing antioxidant response as the abundance decreases and the simulated environment’s pH increases. The critical point that can be considered to demonstrate the modulatory role was at about five months of consumption, identifying a dysbiotic pattern characteristic of the initiation of degenerative pathologies. This innovative study opened the interest for further research through a combination administration with classic sweeteners (sugar), other synthetic or natural sweeteners, prebiotics, and/or probiotics.

## Data Availability

Not applicable.

## Supplementary Materials

The following are available online at https://www.mdpi.com/2076-2607/9/3/590/s1, Figure S1: Daily analysis of *Lactobacillus* strains after in vitro long-term stevioside consumption, Figure S2: The pH variation in the first month after in vitro long-term stevioside consumption, Figure S3: Principal component analysis (PCA) of metabolic profiles, microbial diversity and antioxidant status after in vitro long-term stevioside consumption, Figure S4: Proportion of variance explained by PCA.
