# Supplementary figures and images for: In Vitro Modulatory Effect of Stevioside, as a Partial Sugar Replacer in Sweeteners, on Human Child Microbiota
Source: Microorganisms. 2021 Mar 13;9(3):590. doi: 10.3390/microorganisms9030590 (PMC8000329; doi:10.3390/microorganisms9030590)

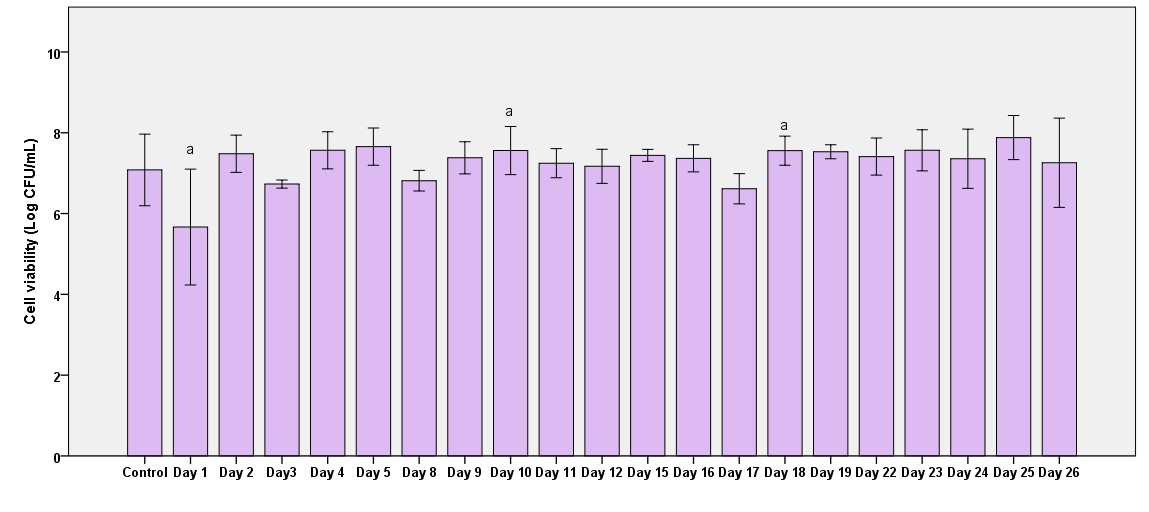

Supplement: Supplementary file 1 [file microorganisms-09-00590-s001.zip › Supplementary Figure S1.jpg]

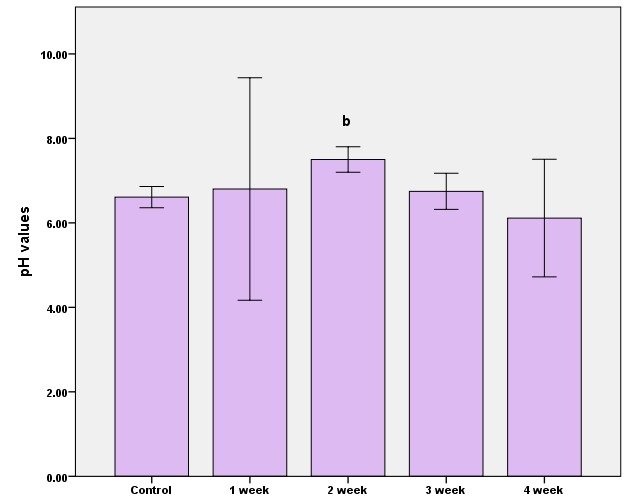

Supplement: Supplementary file 1 [file microorganisms-09-00590-s001.zip › Supplementary Figure S2.jpg]

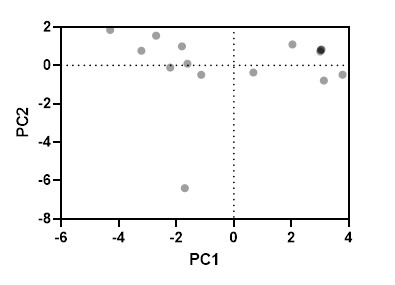

Supplement: Supplementary file 1 [file microorganisms-09-00590-s001.zip › Supplementary Figure S3.jpg]

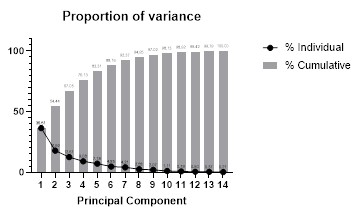

Supplement: Supplementary file 1 [file microorganisms-09-00590-s001.zip › Supplementary Figure S4.jpg]
